# Risk factors for postoperative intensive care unit admission after minimally invasive esophagectomy following neoadjuvant chemoimmunotherapy in patients with esophageal squamous cell carcinoma: a retrospective cohort study

**DOI:** 10.3389/fonc.2026.1917011

**Published:** 2026-08-28

**Authors:** Zhen Chen, Zhi-jing Liu, Qiu-xia Liu, Peng-fei Chen, Yu-cai Jiang

**Affiliations:** 1 Department of Cardiothoracic Surgery, Affiliated Hospital of Putian University, Putian, Fujian, China; 2 Department of Pharmacy, Affiliated Hospital of Putian University, Putian, Fujian, China

**Keywords:** ASA physical status classification, esophageal squamous cell carcinoma, minimally invasive esophagectomy, postoperative intensive care unit admission, prognostic nutritional index

## Abstract

**Introduction:**

Patients with esophageal squamous cell carcinoma (ESCC) who undergo minimally invasive esophagectomy (MIE) after neoadjuvant chemoimmunotherapy (nCIT) and subsequently require postoperative intensive care unit (ICU) admission may experience a greater economic burden and poorer clinical outcomes. This study aimed to identify perioperative factors independently associated with postoperative ICU admission in patients with ESCC treated with nCIT followed by MIE.

**Methods:**

We conducted a retrospective cohort study of patients with ESCC who underwent MIE after nCIT. Baseline demographic characteristics, perioperative variables, postoperative complications, and details of postoperative ICU admission, including incidence, timing, and indications, were systematically collected. Univariable and multivariable logistic regression analyses were performed to identify factors associated with postoperative ICU admission. A nomogram was constructed to provide individualized risk estimates, together with a model-based visualization of the weighted PNI–ASA score and corresponding predicted probability.

**Results:**

A total of 115 eligible patients were included in the final analysis, of whom 25 (21.7%) required postoperative ICU admission. Compared with patients who did not require ICU admission, those admitted to the ICU had significantly higher rates of pulmonary infection (60.0% vs. 11.1%, *p* < 0.001) and respiratory failure (80.0% vs. 41.1%, *p* < 0.001), a longer postoperative hospital stay (26.1 ± 17.1 vs. 18.5 ± 9.1 days, *p* = 0.003), and higher total hospital costs (¥95,519 ± 40,754 vs. ¥63,426 ± 16,895, *p* < 0.001). In the multivariable logistic regression analysis, each unit increase in preoperative prognostic nutritional index (PNI) was associated with lower odds of postoperative ICU admission (OR = 0.84; 95% CI, 0.74–0.94; *p* = 0.002), whereas American Society of Anesthesiologists (ASA) physical status class III was associated with higher odds than class I/II (OR = 7.57; 95% CI, 2.10–27.20; *p* = 0.002). Operative duration was associated with ICU admission in univariable analysis (*p* = 0.024) but not in multivariable analysis (*p* = 0.102). The PNI–ASA nomogram showed acceptable discrimination (apparent AUC = 0.810; optimism-corrected AUC = 0.781).

**Conclusion:**

Lower preoperative PNI and ASA physical status class III were independently associated with postoperative ICU admission in patients with ESCC undergoing nCIT followed by MIE. The PNI–ASA nomogram may support preoperative risk stratification. Although operative duration was not independently associated with ICU admission, it may serve as an intraoperative warning indicator. The dichotomized PNI cutoff derived from the subgroup analyses requires external validation and should not be used as a standalone clinical threshold.

## 1 Introduction

Esophageal cancer is the eighth most commonly diagnosed malignancy and the sixth leading cause of cancer-related mortality worldwide (1). For patients with locally advanced, resectable ESCC, nCIT followed by MIE has emerged as a promising treatment strategy, with recent prospective cohorts demonstrating improved pathological complete response rates and favorable long-term survival outcomes (2–5). Nevertheless, postoperative morbidity remains considerable, and postoperative ICU admission represents an important adverse clinical event with substantial clinical and economic consequences. Patients requiring postoperative ICU admission are at increased risk of in-hospital mortality, prolonged hospitalization, and markedly higher healthcare expenditures (6–11).

The PNI, calculated as serum albumin concentration (g/L) + 5 × total lymphocyte count (×10^9^/L), is an integrated biomarker of host nutritional status and systemic immune competence (12, 13). The ASA Physical Status Classification System provides a standardized and validated assessment of baseline physiological reserve and systemic disease burden (14, 15). Although both indicators have been independently associated with postoperative complications in various surgical populations, their combined discriminatory performance for predicting postoperative ICU admission in patients undergoing nCIT followed by MIE remains unclear (16–18).

Accordingly, this retrospective cohort study aimed to identify preoperative factors independently associated with postoperative ICU admission in patients with ESCC undergoing nCIT followed by MIE and to develop a clinically applicable nomogram for individualized preoperative risk stratification based on readily available preoperative variables. We also explored the association between intraoperative variables and postoperative ICU admission.

## 2 Methods

### 2.1 Study design and setting

This single-center retrospective cohort study was conducted at the Affiliated Hospital of Putian University. We screened 123 consecutive patients who underwent MIE for thoracic ESCC between January 2019 and December 2025. The study protocol was reviewed and approved by the Institutional Review Board of the Affiliated Hospital of Putian University. The requirement for informed consent was waived because of the retrospective, non-interventional nature of the study and the complete anonymization of patient data.

### 2.2 Participants

Eligibility was assessed among the 123 patients screened during the study period.

The inclusion criteria were as follows (1): histopathologically confirmed thoracic ESCC; (2) clinical stage cT1–2N+M0 or cT3–4aNxM0 according to the eighth edition of the American Joint Committee on Cancer/Union for International Cancer Control (AJCC/UICC) TNM staging system; (3) age ≥18 years; (4) Eastern Cooperative Oncology Group performance status of 0 or 1; (5) no previous antitumor treatment for esophageal cancer, including radiotherapy, chemotherapy, or surgery; (6) receipt of at least two cycles of nCIT consisting of platinum-based chemotherapy combined with a programmed cell death protein 1 (PD-1) or programmed death-ligand 1 (PD-L1) inhibitor; (7) completion of R0 McKeown MIE with thoracic and abdominal lymphadenectomy within 6–8 weeks after nCIT; and (8) availability of complete preoperative laboratory data, including serum albumin concentration and absolute lymphocyte count, imaging reports, and structured perioperative records.

**Figure 1:**
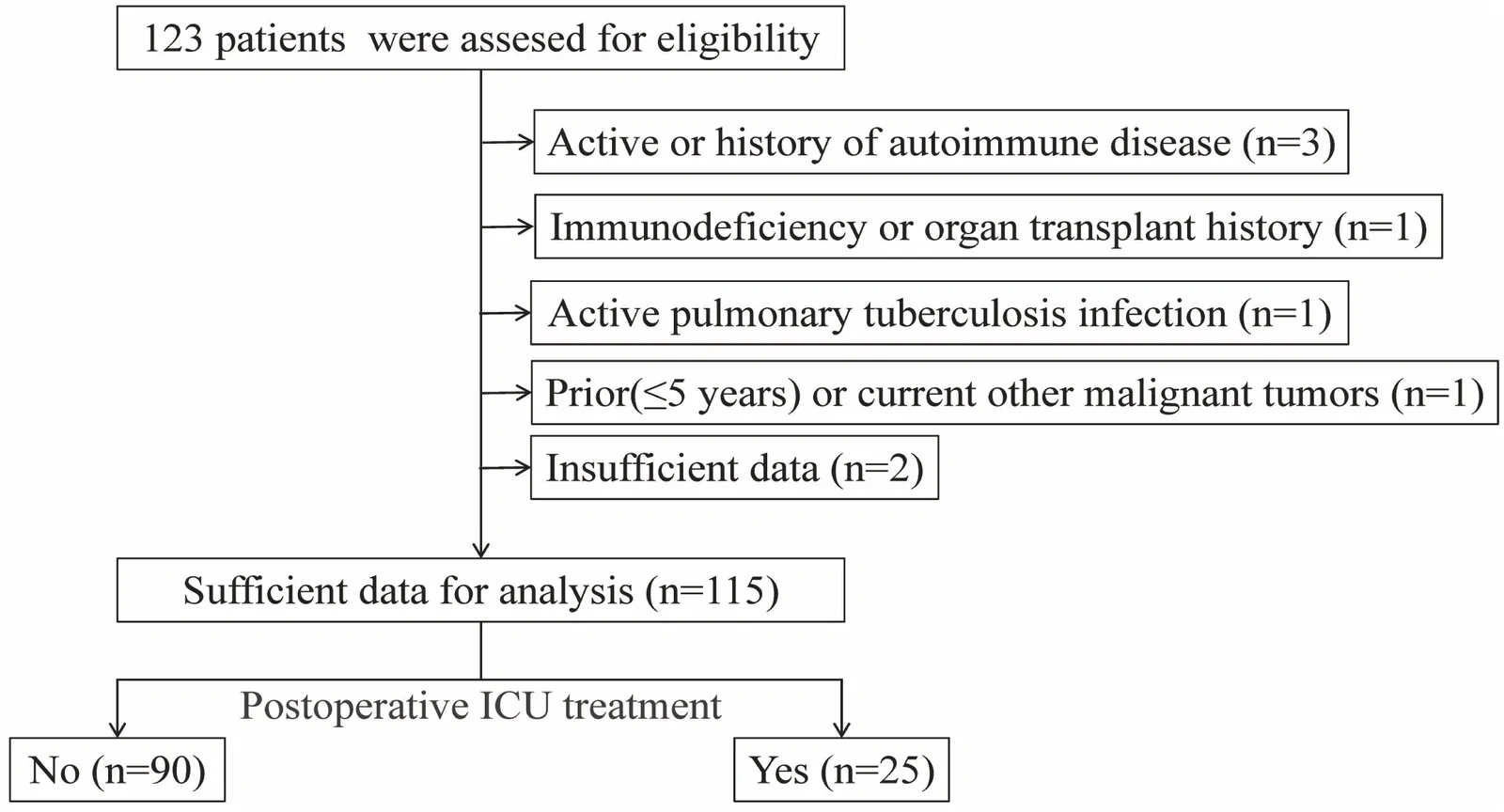
Flow diagram of patient selection. MIE, minimally invasive esophagectomy; ICU, intensive care unit.

The exclusion criteria were as follows: (1) non-squamous histology, including adenocarcinoma or small-cell carcinoma; (2) tumor located in the cervical or abdominal esophagus; (3) absence of neoadjuvant therapy; (4) neoadjuvant treatment without immunotherapy, including chemotherapy alone or chemoradiotherapy; (5) a non-MIE procedure, including open esophagectomy, a hybrid approach, or palliative resection, or failure to achieve R0 resection; (6) active or previous autoimmune disease; (7) immunodeficiency or a history of organ transplantation; (8) active pulmonary tuberculosis; (9) a previous malignancy within 5 years or a concurrent malignancy; and (10) missing key variables required for prognostic modeling, including the components of preoperative PNI, ASA physical status class, or operative duration. After application of these criteria, 115 patients with thoracic ESCC who received nCIT followed by MIE were included in the final analysis. Of these, 25 (21.7%) required postoperative ICU admission and 90 (78.3%) did not. The patient selection process is summarized in **Figure 1**.

Postoperative ICU admission was defined as any transfer to the ICU after surgery. At our center, patients undergoing MIE after nCIT are routinely returned to the cardiothoracic ward, and ICU transfer is reserved for specific indications; therefore, all 25 ICU admissions in this cohort were unplanned. The indications were as follows: (1) acute respiratory failure requiring invasive ventilation or high-flow nasal cannula oxygen therapy beyond the capacity of standard ward care, including difficult or failed extubation in the operating room or post-anesthesia care unit; (2) persistent hemodynamic instability unresponsive to initial fluid resuscitation and requiring vasopressor support; or (3) an acute life-threatening cardiorespiratory or cerebrovascular event, such as myocardial infarction, massive pulmonary embolism, or ischemic stroke, requiring continuous multimodal monitoring and intervention. These indications remained unchanged throughout the study period. All postoperative ICU admissions were jointly reviewed and confirmed by senior cardiothoracic surgeons and intensivists.

### 2.3 Surgical procedure

All patients underwent a standardized McKeown MIE procedure comprising thoracoscopic mobilization of the thoracic esophagus, laparoscopic gastric mobilization and gastric conduit formation, and cervical esophagogastric anastomosis through the posterior mediastinal route. Routine intraoperative measures included placement of a nasogastric decompression tube and a nasojejunal feeding tube under direct visualization. Surgical quality was ensured by achieving R0 resection with proximal and distal tumor margins of at least 5 cm and performing complete thoracic and abdominal lymphadenectomy in accordance with IASLC/ITMIG guidelines. All procedures were performed by surgeons with at least 5 years of dedicated experience in minimally invasive upper gastrointestinal oncologic surgery.

### 2.4 Intraoperative anesthesia monitoring

Lung isolation was achieved using a single-lumen endotracheal tube combined with a bronchial blocker. An intraoperative lung-protective ventilation strategy was used, targeting a tidal volume of 6–8 mL/kg predicted body weight and an initial positive end-expiratory pressure (PEEP) of 5 cmH_2_O. Epidural analgesia was not administered in this cohort. Preoperative risk was assessed using the ASA Physical Status Classification System and the Charlson Comorbidity Index, as summarized in **Supplementary Table 1**.

### 2.5 Statistical analysis

A panel of preoperative systemic inflammatory and nutritional biomarkers, including the neutrophil-to-lymphocyte ratio, platelet-to-lymphocyte ratio, monocyte-to-lymphocyte ratio, systemic immune-inflammation index, and PNI, was evaluated for univariable associations with postoperative ICU admission (**Supplementary Table 2**). Based on its performance in the univariable analysis and its biological plausibility, PNI was prioritized for inclusion in the multivariable model, consistent with previous evidence in upper gastrointestinal oncology (19, 20).

The primary endpoint was postoperative ICU admission. Categorical variables were compared using the chi-square test or Fisher’s exact test, as appropriate. Continuous variables were compared using Student’s t-test or the Mann–Whitney U test, depending on their distribution.

#### 2.5.1 Model development and presentation

To improve the transparency and reproducibility of model development, we explicitly defined the hierarchical relationship among the analytical components presented in this study.

#### 2.5.2 Primary model

Variable selection followed a two-step approach. First, variables with *p* < 0.05 in the univariable logistic regression analysis (age ≥65 years, PNI, ASA class III, and operative duration) were entered into the core multivariable model (**Table 1**, Model 1). Second, additional covariates were selected *a priori* on the basis of previous literature and clinical relevance and were included in the exploratory adjustment models (**Table 1**, Models 2 and 3). Multicollinearity was assessed using the variance inflation factor (VIF), with values >5 and >10 indicating moderate and severe multicollinearity, respectively. The highest VIF was 1.603, indicating no substantial multicollinearity (**Supplementary Table 3**). The final model (Model 4) included preoperative PNI, modeled as a continuous variable, and ASA physical status, modeled as class III versus class I/II. These variables were retained because their associations remained statistically significant in the univariable and multivariable analyses (all *p* < 0.05) and because of their clinical relevance (11, 18). Operative duration, although associated with the outcome in the univariable analysis (*p* = 0.024), was not statistically significant in the fully adjusted multivariable model (*p* = 0.102) and was therefore not included in the predictive nomogram. The PNI–ASA model was selected as the primary model for clinical presentation because it includes only preoperatively available variables and is therefore suitable for preoperative risk communication.

**Table 1:** Univariable and multivariable logistic regression analyses of factors associated with postoperative ICU admission, including the final parsimonious PNI–ASA model.

Variables	Univariable	Multivariable 1	Multivariable 2	Multivariable 3	Multivariable 4 (Final PNI–ASA model)
	OR (95% CI), *p*	OR (95% CI), *p*	OR (95% CI), *p*	OR (95% CI), *p*	OR (95% CI), *p*
Sex (reference: male)					
Female	0.73 (0.25–2.16), 0.568		0.76 (0.19–2.98), 0.690	0.86 (0.21–3.52), 0.830	
Age (reference: <65 years)					
≥65 years	3.34 (1.06–10.55), 0.040	3.22 (0.80–13.02), 0.101	3.29 (0.74–14.70), 0.118	3.00 (0.66–13.65), 0.154	
BMI (continuous)	1.08 (0.94–1.25), 0.276		1.20 (0.98–1.48), 0.078	1.21 (0.98–1.49), 0.083	
Smoking (reference: no)					
Yes	0.44 (0.14–1.42), 0.171				
Alcohol use (reference: no)					
Yes	0.29 (0.06–1.31), 0.107				
Hypertension (reference: no)					
Yes	1.07 (0.40–2.88), 0.894			1.07 (0.23–4.89), 0.935	
Diabetes mellitus (reference: no)					
Yes	0.38 (0.05–3.11), 0.363			0.44 (0.03–5.96), 0.538	
Immunotherapy agent (reference: PD-1)					
PD-L1	1.13 (0.45–2.84), 0.803			1.76 (0.47–6.60), 0.404	
Neoadjuvant treatment cycles (reference: 2 cycles)					
3 or 4 cycles	1.80 (0.56–5.76), 0.325			1.25 (0.25–6.27), 0.783	
PNI (continuous)	0.83 (0.75–0.93), 0.001	0.85 (0.76–0.96), 0.009	0.84 (0.74–0.95), 0.006	0.84 (0.74–0.96), 0.009	0.84 (0.74–0.94), 0.002
ASA physical status (reference: class I/II)					
Class III	7.88 (2.46–25.20), 0.001	7.71 (2.03–29.28), 0.003	9.45 (2.19–40.88), 0.003	10.22 (2.14–48.88), 0.004	7.57 (2.10–27.20), 0.002
FEV1, L (continuous)	0.62 (0.31–1.25), 0.182			0.85 (0.31–2.29), 0.746	
EF, % (continuous)	0.97 (0.88–1.06), 0.508				
Tumor location (reference: middle segment)					
Upper or lower segment	1.18 (0.47–2.99), 0.723				
Operative duration, min (continuous)	1.01 (1.00–1.01), 0.024	1.01 (1.00–1.02), 0.015	1.01 (1.00–1.01), 0.072	1.01 (1.00–1.02), 0.102	
Intraoperative blood loss (reference: <500 mL)					
≥500 mL	2.02 (0.75–5.45), 0.165		1.36 (0.97–1.91), 0.076	1.39 (0.97–2.01), 0.073	

Model 1 included age ≥65 years, PNI, ASA class III, and operative duration, which were statistically significant in the univariable analyses.

Model 2 additionally adjusted for sex, BMI, and intraoperative blood loss.

Model 3 further adjusted for hypertension, diabetes, immunotherapy type, number of neoadjuvant treatment cycles, and FEV1.

Model 4 was the final parsimonious prediction model and included only preoperative PNI and ASA physical status. Model 4 was used to construct the PNI–ASA nomogram.

Empty cells indicate that the corresponding variable was not included in the respective model. ORs and 95% CIs were estimated using logistic regression, and *p* values were two-sided. BMI, body mass index; PNI, prognostic nutritional index; ASA, American Society of Anesthesiologists; FEV1, forced expiratory volume in 1 second; EF, ejection fraction.

Empty cells indicate that the variable was not included in the respective multivariable model. Confidence intervals were calculated using the Wald method, and *p* values were two-sided.

#### 2.5.3 Nomogram

**Figure 2:**
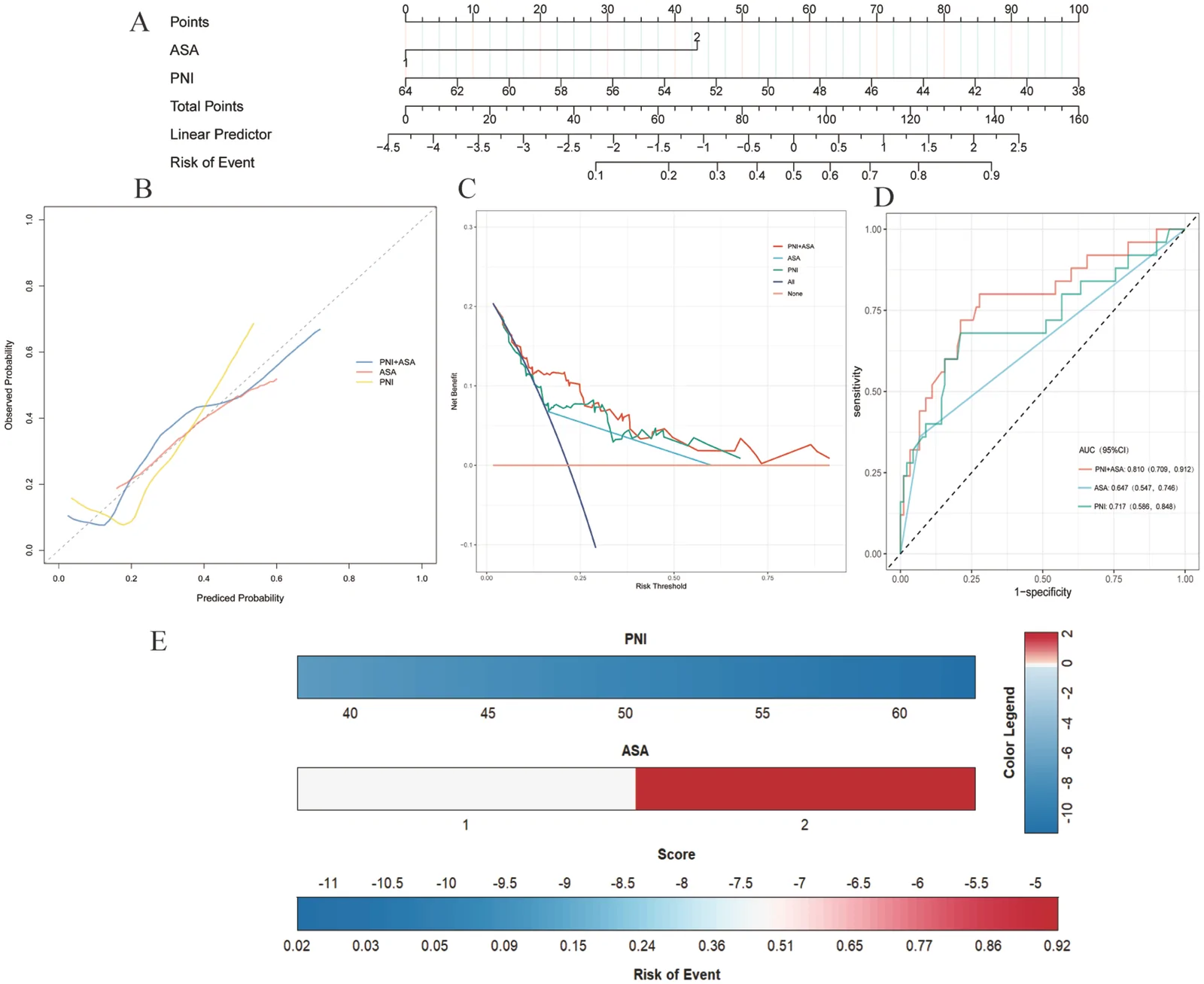
Nomogram and performance evaluation of the PNI–ASA model. **(A)** Nomogram incorporating PNI and ASA physical status. PNI ranges from 38 to 64, and ASA physical status is coded as class I/II = 1 and class III = 2. Total points are mapped to a linear predictor ranging from −4.5 to +2.5 and to the corresponding predicted probability of postoperative ICU admission. **(B)** Calibration curve comparing predicted and observed probabilities of postoperative ICU admission (intercept = −0.00; slope = 1.00; Hosmer–Lemeshow *p* = 0.821). **(C)** Decision curve analysis showing the estimated net benefit of the PNI–ASA model across threshold probabilities of 0.00–0.50. **(D)** Receiver operating characteristic curves for the PNI–ASA model (AUC = 0.810; 95% CI, 0.709–0.912), ASA alone (AUC = 0.647; 95% CI, 0.547–0.746), and PNI alone (AUC = 0.717; 95% CI, 0.586–0.846). **(E)** Model-based visualization of the weighted PNI–ASA score and the corresponding predicted probability of postoperative ICU admission. The displayed score represents the weighted contribution of PNI and ASA physical status to the model linear predictor, excluding the intercept. Lower, more negative scores indicate lower predicted risk, whereas higher, less negative scores indicate higher predicted risk. In this panel, ASA category 1 denotes class I/II and category 2 denotes class III. This visualization does not constitute a separate integer-based scoring system.

The nomogram (**Figure 2A**) provides a graphical representation of the primary logistic regression model. It was constructed by linearly transforming the regression coefficients for PNI and ASA class onto a 0–100-point scale, from which the predicted probability of postoperative ICU admission can be obtained. The nomogram incorporates preoperative PNI and ASA physical status to provide an intuitive graphical representation of postoperative ICU admission risk and does not constitute a separate model. Areas under the receiver operating characteristic curves (AUCs) were compared using the DeLong test, with the results presented in **Supplementary Table 4**.

Model-based risk-score visualization. To facilitate interpretation of the final PNI–ASA model, we generated a graphical visualization of the weighted contributions of continuous PNI and ASA physical status to the predicted probability of postoperative ICU admission. The displayed model score represents the linear combination of PNI and ASA physical status weighted by their corresponding regression coefficients, excluding the model intercept. The predicted probability was obtained from the complete linear predictor, including the intercept, using the inverse-logit transformation. This visualization did not constitute a separate clinical scoring system and did not introduce additional predictors or cutoff values.

#### 2.5.4 Internal validation

The nomogram was internally validated and corrected for optimism using 1,000 bootstrap iterations (**Supplementary Table 5**). In each iteration, a bootstrap sample of the same size as the original dataset (n = 115) was drawn with replacement. The full model-development process, including the same variable-selection and logistic regression procedures, was repeated in each bootstrap sample. Discrimination, quantified using the AUC, was assessed in each bootstrap sample and when the resulting model was applied to the original dataset. Optimism was calculated as the mean difference between these AUCs and was subtracted from the apparent performance of the model fitted to the full original cohort to obtain the optimism-corrected estimate. Calibration slopes were corrected similarly. This bootstrap-based optimism-correction procedure followed the method described by Harrell and colleagues (21).

#### 2.5.5 Binary risk classification for subgroup analyses

**Figure 3:**
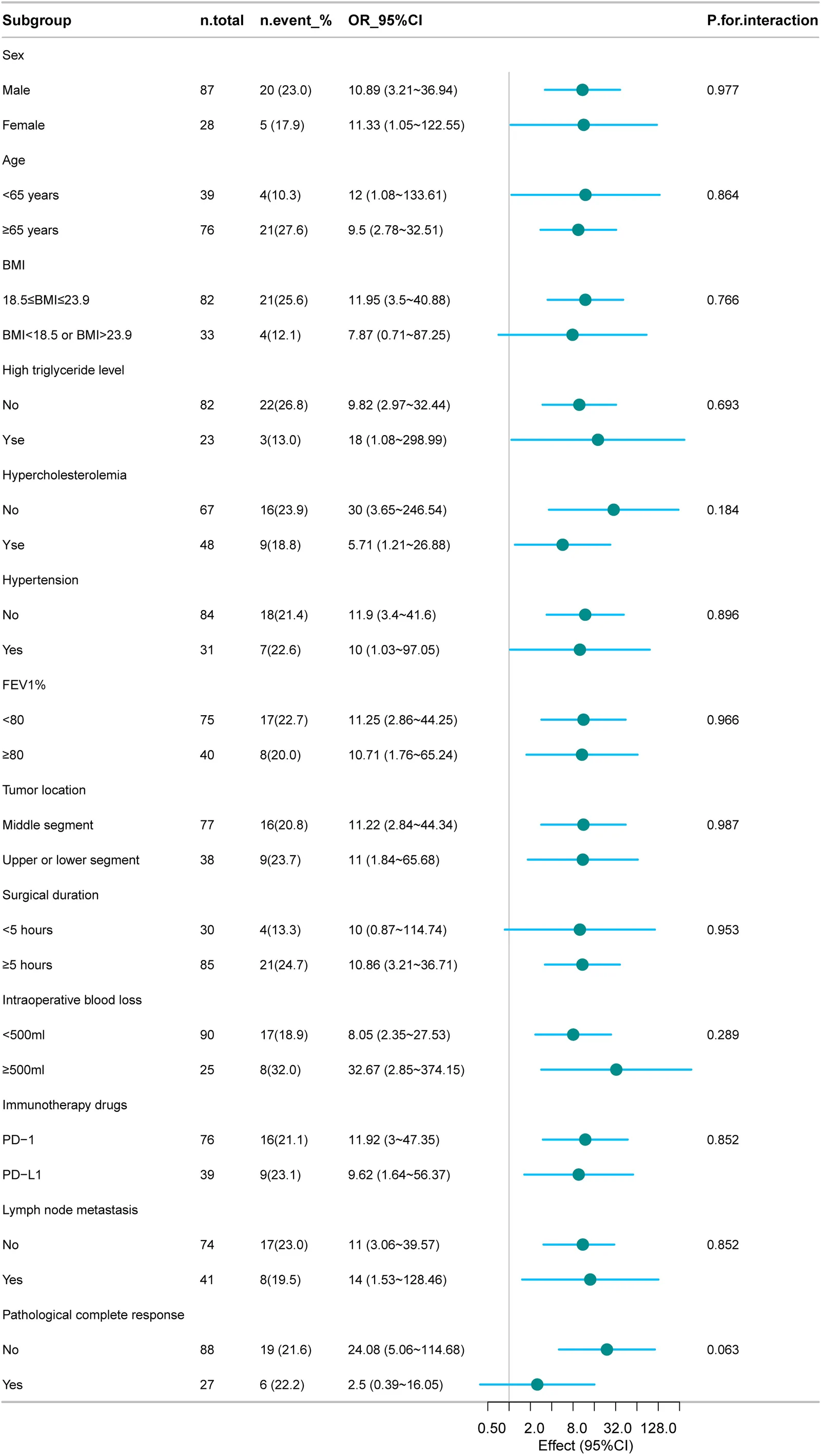
Forest plot of exploratory subgroup analyses based on the binary PNI–ASA risk classification. The plot shows odds ratios (ORs) and 95% confidence intervals (CIs) for postoperative ICU admission in the high-risk group versus the low-risk group across predefined subgroups. The high-risk group comprised patients with PNI <47.225, ASA class III, or both; the low-risk group comprised patients with PNI ≥47.225 and ASA class I/II. The size of each square is proportional to the inverse variance of the log OR. Horizontal lines represent 95% CIs, and the vertical dashed line indicates an OR of 1.0. Interaction *p* values are shown on the right.

For the subgroup analyses, PNI was dichotomized at the Youden index-derived cutoff of 47.225 (**Supplementary Table 6**), and ASA physical status was dichotomized as class III versus class I/II. Patients with both PNI ≥47.225 and ASA class I/II were classified as low risk, whereas those with PNI <47.225, ASA class III, or both were classified as high risk. This binary classification was used exclusively to evaluate the consistency of the association across patient strata (**Figure 3**, **Table 2**) and was not considered an independent primary prediction model.

**Table 2:** Exploratory subgroup analyses of the association between the binary PNI–ASA risk classification and postoperative ICU admission.

Subgroup	Variable	Risk group	Total n	Events, n (%)	OR (95% CI)	*p* for interaction
Sex						0.977
	Male	Low risk	53	4 (7.5)	1 (Reference)	
		High risk	34	16 (47.1)	10.89 (3.21–36.94)	
	Female	Low risk	18	1 (5.6)	1 (Reference)	
		High risk	10	4 (40.0)	11.33 (1.05–122.55)	
Age						0.864
	<65 years	Low risk	29	1 (3.4)	1 (Reference)	
		High risk	10	3 (30.0)	12.00 (1.08–133.61)	
	≥65 years	Low risk	42	4 (9.5)	1 (Reference)	
		High risk	34	17 (50.0)	9.50 (2.78–32.51)	
BMI						0.766
	18.5 ≤ BMI ≤ 23.9	Low risk	49	4 (8.2)	1 (Reference)	
		High risk	33	17 (51.5)	11.95 (3.50–40.88)	
	BMI < 18.5 or > 23.9	Low risk	22	1 (4.5)	1 (Reference)	
		High risk	11	3 (27.3)	7.87 (0.71–87.25)	
High triglyceride level						0.693
	No	Low risk	52	4 (7.7)	1 (Reference)	
		High risk	40	18 (45.0)	9.82 (2.97–32.44)	
	Yes	Low risk	19	1 (5.3)	1 (Reference)	
		High risk	4	2 (50.0)	18.00 (1.08–298.99)	
Hypercholesterolemia						0.184
	No	Low risk	35	1 (2.9)	1 (Reference)	
		High risk	32	15 (46.9)	30.00 (3.65–246.54)	
	Yes	Low risk	36	4 (11.1)	1 (Reference)	
		High risk	12	5 (41.7)	5.71 (1.21–26.88)	
Hypertension						0.896
	No	Low risk	55	4 (7.3)	1 (Reference)	
		High risk	29	14 (48.3)	11.90 (3.40–41.60)	
	Yes	Low risk	16	1 (6.2)	1 (Reference)	
		High risk	15	6 (40.0)	10.00 (1.03–97.05)	
FEV1%						0.966
	<80	Low risk	44	3 (6.8)	1 (Reference)	
		High risk	31	14 (45.2)	11.25 (2.86–44.25)	
	≥80	Low risk	27	2 (7.4)	1 (Reference)	
		High risk	13	6 (46.2)	10.71 (1.76–65.24)	
Tumor location						0.987
	Middle segment	Low risk	47	3 (6.4)	1 (Reference)	
		High risk	30	13 (43.3)	11.22 (2.84–44.34)	
	Upper or lower segment	Low risk	24	2 (8.3)	1 (Reference)	
		High risk	14	7 (50.0)	11.00 (1.84–65.68)	
Surgical duration						0.953
	<5 hours	Low risk	21	1 (4.8)	1 (Reference)	
		High risk	9	3 (33.3)	10.00 (0.87–114.74)	
	≥5 hours	Low risk	50	4 (8.0)	1 (Reference)	
		High risk	35	17 (48.6)	10.86 (3.21–36.71)	
Intraoperative blood loss						0.289
	<500 mL	Low risk	56	4 (7.1)	1 (Reference)	
		High risk	34	13 (38.2)	8.05 (2.35–27.53)	
	≥500 mL	Low risk	15	1 (6.7)	1 (Reference)	
		High risk	10	7 (70.0)	32.67 (2.85–374.15)	
Immunotherapy drug						0.852
	PD-1	Low risk	47	3 (6.4)	1 (Reference)	
		High risk	29	13 (44.8)	11.92 (3.00–47.35)	
	PD-L1	Low risk	24	2 (8.3)	1 (Reference)	
		High risk	15	7 (46.7)	9.62 (1.64–56.37)	
Lymph node metastasis						0.852
	No	Low risk	48	4 (8.3)	1 (Reference)	
		High risk	26	13 (50.0)	11.00 (3.06–39.57)	
	Yes	Low risk	23	1 (4.3)	1 (Reference)	
		High risk	18	7 (38.9)	14.00 (1.53–128.46)	
Pathological complete response						0.063
	No	Low risk	53	2 (3.8)	1 (Reference)	
		High risk	35	17 (48.6)	24.08 (5.06–114.68)	
	Yes	Low risk	18	3 (16.7)	1 (Reference)	
		High risk	9	3 (33.3)	2.50 (0.39–16.05)	

*ICU, intensive care unit; OR, odds ratio; CI, confidence interval; BMI, body mass index; FEV1, forced expiratory volume in 1 second; PD-1, programmed cell death protein 1; PD-L1, programmed death-ligand 1.

*The low-risk group comprised patients with both PNI ≥47.225 and ASA physical status class I/II. The high-risk group comprised patients with PNI <47.225, ASA physical status class III, or both. This binary classification was used exclusively for exploratory subgroup analyses and was not used to construct or validate the primary PNI–ASA model.

#### 2.5.6 Software and reproducibility

All logistic regression models were fitted using the glm function in R version 4.2.3 (R Foundation for Statistical Computing, Vienna, Austria) (22, 23). The nomogram was constructed using the rms package. The weighted model score displayed in **Figure 2E** was calculated from the regression coefficients of PNI and ASA physical status.

## 3 Results

### 3.1 Patient characteristics

A total of 115 patients were included in the final analysis, of whom 25 (21.7%) required postoperative ICU admission (**Figure 1**). Consistent with institutional practice, none of these ICU admissions were planned preoperatively; all were unplanned transfers prompted by postoperative complications. Compared with patients who did not require ICU admission, those who did were older (70.0 ± 7.4 vs. 65.2 ± 8.2 years, *p* = 0.010), were more likely to be aged ≥65 years (84.0% vs. 61.1%, *p* = 0.032), had a higher prevalence of ASA class III (36.0% vs. 6.7%, *p* < 0.001), and had a lower preoperative PNI (47.1 ± 5.5 vs. 51.2 ± 4.6, *p* < 0.001). They also had higher incidences of pulmonary infection (60.0% vs. 11.1%, *p* < 0.001), respiratory failure (80.0% vs. 41.1%, *p* < 0.001), and pleural effusion (24.0% vs. 5.6%, *p* = 0.013). Accordingly, these patients had longer postoperative hospital stays (26.1 ± 17.1 vs. 18.5 ± 9.1 days, *p* = 0.003) and total hospital stays (36.4 ± 16.2 vs. 27.3 ± 11.3 days, *p* = 0.002), as well as higher total hospital costs (¥95,519 ± 40,754 vs. ¥63,426 ± 16,895, *p* < 0.001). In-hospital mortality was numerically higher in the ICU-admission group than in the non-ICU-admission group (8.0% vs. 1.1%), although the difference was not statistically significant (*p* = 0.119). Baseline and perioperative characteristics are summarized in **Table 3**. The median operative duration for the entire cohort was 346.0 min (IQR, 295.0–393.5 min). Patients who required postoperative ICU admission had a longer median operative duration than those who did not (379.8 min [IQR, 325.0–425.0] vs. 340.5 min [IQR, 287.0–381.0], *p* = 0.018).

**Table 3:** Baseline and perioperative characteristics of patients with and without postoperative ICU admission after nCIT followed by MIE.

Variable	Total (n = 115)	No ICU admission (n = 90)	ICU admission (n = 25)	*p* value
Sex, n (%)				0.567
Male	87 (75.7)	67 (74.4)	20 (80.0)	
Female	28 (24.3)	23 (25.6)	5 (20.0)	
Age, years, mean ± SD	66.3 ± 8.2	65.2 ± 8.2	70.0 ± 7.4	0.010
Age group, n (%)				0.032
<65 years	39 (33.9)	35 (38.9)	4 (16.0)	
≥65 years	76 (66.1)	55 (61.1)	21 (84.0)	
Smoking, n (%)				0.163
No	84 (73.0)	63 (70.0)	21 (84.0)	
Yes	31 (27.0)	27 (30.0)	4 (16.0)	
Drinking, n (%)				0.090
No	92 (80.0)	69 (76.7)	23 (92.0)	
Yes	23 (20.0)	21 (23.3)	2 (8.0)	
Immunotherapy drug, n (%)				0.803
PD-1	76 (66.1)	60 (66.7)	16 (64.0)	
PD-L1	39 (33.9)	30 (33.3)	9 (36.0)	
Neoadjuvant cycles, n (%)				0.335
2 cycles	99 (86.1)	79 (87.8)	20 (80.0)	
3 or 4 cycles	16 (13.9)	11 (12.2)	5 (20.0)	
ASA physical status, n (%)				<0.001
Class I/II	100 (87.0)	84 (93.3)	16 (64.0)	
Class III	15 (13.0)	6 (6.7)	9 (36.0)	
BMI, kg/m², mean ± SD	21.8 ± 3.1	21.7 ± 3.1	22.4 ± 3.0	0.277
Hypertension, n (%)				0.894
No	84 (73.0)	66 (73.3)	18 (72.0)	
Yes	31 (27.0)	24 (26.7)	7 (28.0)	
Diabetes mellitus, n (%)				0.688
No	105 (91.3)	81 (90.0)	24 (96.0)	
Yes	10 (8.7)	9 (10.0)	1 (4.0)	
aCCI, n (%)				0.074
0–1 score	3 (2.6)	3 (3.3)	0 (0)	
2–3 scores	53 (46.1)	46 (51.1)	7 (28.0)	
4–5 scores	56 (48.7)	38 (42.2)	18 (72.0)	
≥6 scores	3 (2.6)	3 (3.3)	0 (0)	
PNI, mean ± SD	50.3 ± 5.1	51.2 ± 4.6	47.1 ± 5.5	<0.001
FEV1, L, mean ± SD	2.5 ± 0.7	2.6 ± 0.6	2.4 ± 0.8	0.181
EF, %, mean ± SD	64.6 ± 4.7	64.8 ± 4.2	64.1 ± 6.3	0.510
Operative duration, n (%)				0.194
≤5 hours	30 (26.1)	26 (28.9)	4 (16.0)	
>5 hours	85 (73.9)	64 (71.1)	21 (84.0)	
Intraoperative blood loss, n (%)				0.160
<500 mL	90 (78.3)	73 (81.1)	17 (68.0)	
≥500 mL	25 (21.7)	17 (18.9)	8 (32.0)	
Tumor location, n (%)				0.422
Middle segment	77 (67.0)	61 (67.8)	16 (64.0)	
Upper or lower segment	38 (33.0)	29 (32.2)	9 (36.0)	
T stage, n (%)				0.835
0	34 (29.6)	25 (27.8)	9 (36.0)	
1a	11 (9.6)	8 (8.9)	3 (12.0)	
1b	14 (12.2)	11 (12.2)	3 (12.0)	
2	32 (27.8)	27 (30.0)	5 (20.0)	
3	24 (20.9)	19 (21.1)	5 (20.0)	
N stage, n (%)				0.720
0	74 (64.3)	57 (63.3)	17 (68.0)	
1	29 (25.2)	22 (24.4)	7 (28.0)	
2	11 (9.6)	10 (11.1)	1 (4.0)	
3	1 (0.9)	1 (1.1)	0 (0)	
pCR, n (%)				0.945
No	88 (76.5)	69 (76.7)	19 (76.0)	
Yes	27 (23.5)	21 (23.3)	6 (24.0)	
MPR, n (%)				0.894
No	84 (73.0)	66 (73.3)	18 (72.0)	
Yes	31 (27.0)	24 (26.7)	7 (28.0)	
Anastomotic fistula, n (%)				0.688
No	105 (91.3)	81 (90.0)	24 (96.0)	
Yes	10 (8.7)	9 (10.0)	1 (4.0)	
Pneumonia, n (%)				<0.001
No	90 (78.3)	80 (88.9)	10 (40.0)	
Yes	25 (21.7)	10 (11.1)	15 (60.0)	
Respiratory failure, n (%)				<0.001
No	58 (50.4)	53 (58.9)	5 (20.0)	
Yes	57 (49.6)	37 (41.1)	20 (80.0)	
Pleural effusion, n (%)				0.013
No	104 (90.4)	85 (94.4)	19 (76.0)	
Yes	11 (9.6)	5 (5.6)	6 (24.0)	
Arrhythmia, n (%)				0.631
No	87 (75.7)	69 (76.7)	18 (72.0)	
Yes	28 (24.3)	21 (23.3)	7 (28.0)	
Perioperative mortality, n (%)				0.119
No	112 (97.4)	89 (98.9)	23 (92.0)	
Yes	3 (2.6)	1 (1.1)	2 (8.0)	
Postoperative hospital stay, days, mean ± SD	20.1 ± 11.7	18.5 ± 9.1	26.1 ± 17.1	0.003
Total hospital stay, days, mean ± SD	29.3 ± 13.0	27.3 ± 11.3	36.4 ± 16.2	0.002
Total hospital cost, ¥, mean ± SD	70,402.8 ± 27,372.5	63,426.1 ± 16,894.5	95,519.1 ± 40,753.8	<0.001

*ICU, intensive care unit; SD, standard deviation; ASA, American Society of Anesthesiologists; BMI, body mass index; aCCI, age-adjusted Charlson Comorbidity Index; PNI, prognostic nutritional index; FEV1, forced expiratory volume in 1 second; EF, ejection fraction; pCR, pathological complete response; MPR, major pathological response.

Of the 25 patients admitted to the ICU, 15 (60.0%) were admitted within 72 hours after surgery. The median time to ICU admission was postoperative day 1 (IQR, 0–3 days; range, 0–27 days). The latest ICU admission occurred on postoperative day 27 in a patient who developed respiratory failure secondary to cervical anastomotic leakage. The median ICU length of stay was 91.0 hours (IQR, 35.5–212.5 hours; range, 12.5–264.0 hours). Reintubation was required in 12 patients (48.0%), and the median duration of tracheal intubation was 4 days (IQR, 2–7 days; range, 1–22 days). The indications for postoperative ICU admission and the corresponding ICU lengths of stay are summarized in **Table 4**. The detailed temporal course of each ICU-admitted patient is presented in **Supplementary Table 7**.

**Table 4:** Indications for postoperative ICU admission and corresponding ICU lengths of stay.

Indication for ICU admission	n (%)	ICU length of stay, h, median (IQR)
Pneumonia	4 (16)	140.2 (71.8–197.4)
Respiratory failure	4 (16)	187.2 (97.5–264.0)
Pneumonia plus respiratory failure	8 (32)	209.0 (112.2–256.8)
Pulmonary embolism*	1 (4)	35.5†
Hyperkalemia	1 (4)	23.5†
Acute ischemic intestinal necrosis	1 (4)	12.5†
Difficulty with tracheal extubation after anesthesia	3 (12)	19.0 (16.5–21.0)
Difficulty with tracheal extubation after anesthesia plus pneumonia	3 (12)	65.0 (54.0–78.0)

*Pulmonary artery embolism is referred to as pulmonary embolism for brevity.

†For a single patient (n = 1), the individual value is presented without an IQR.

### 3.2 Risk factors for postoperative ICU admission

Univariable logistic regression identified four variables significantly associated with postoperative ICU admission: age ≥65 years (OR = 3.34; 95% CI, 1.06–10.55; *p* = 0.040), PNI (OR = 0.83 per unit increase; 95% CI, 0.75–0.93; *p* = 0.001), ASA class III (OR = 7.88; 95% CI, 2.46–25.20; *p* < 0.001), and operative duration (OR = 1.01 per minute; 95% CI, 1.00–1.01; *p* = 0.024). As shown in **Table 1**, higher PNI was associated with lower odds of postoperative ICU admission, whereas older age, higher ASA class, and longer operative duration were associated with higher odds.

To assess the robustness of these associations, we constructed three progressively adjusted multivariable models (**Table 1**, Models 1–3). The direction and magnitude of the associations for PNI and ASA physical status were broadly consistent across these models. In the final parsimonious model (Model 4), each one-point increase in preoperative PNI was associated with lower odds of postoperative ICU admission (OR = 0.84; 95% CI, 0.74–0.94; *p* = 0.002), whereas ASA class III was associated with higher odds than ASA class I/II (OR = 7.57; 95% CI, 2.10–27.20; *p* = 0.002).

In the most fully adjusted exploratory model (Model 3), neither operative duration (OR = 1.01 per minute; 95% CI, 1.00–1.02; *p* = 0.102) nor age ≥65 years (*p* = 0.154) remained statistically significant. Collinearity diagnostics for the extended model showed no substantial multicollinearity among the included covariates (maximum VIF = 1.603; **Supplementary Table 3**).

Based on these findings, we constructed a parsimonious predictive model incorporating only the preoperatively available predictors PNI and ASA class to maximize its clinical utility for preoperative risk stratification.

### 3.3 Prediction model for postoperative ICU admission

The nomogram provides a graphical representation of the final parsimonious PNI–ASA model (**Figure 2A**). PNI was entered as a continuous variable ranging from 38 to 64, whereas ASA physical status was coded as class I/II = 1 and class III = 2. The total point score (0–160) was linearly transformed to a log-odds scale ranging from −4.5 to +2.5, corresponding to predicted probabilities of postoperative ICU admission ranging from 0.10 to 0.90.

The PNI–ASA model demonstrated acceptable discrimination, with an apparent AUC of 0.810 (95% CI, 0.709–0.912). The AUCs for ASA alone and PNI alone were 0.647 (95% CI, 0.547–0.746) and 0.717 (95% CI, 0.586–0.846), respectively (**Supplementary Table 4**). The DeLong test showed that the combined model significantly outperformed ASA alone (*p* = 0.003; AUC difference, 0.163; 95% CI, 0.059–0.263), whereas its performance did not differ significantly from that of PNI alone (*p* = 0.178). After internal validation using 1,000 bootstrap resamples and optimism correction, the optimism-corrected AUC was 0.781 (95% CI, 0.674–0.888), with a corrected calibration slope of 0.94 and an intercept of −0.02. The small difference between the apparent and corrected performance estimates (optimism = 0.029) suggested limited overfitting and acceptable internal validity (**Supplementary Table 5**).

Calibration analysis showed close agreement between predicted and observed probabilities (intercept = −0.00; 95% CI, −0.50 to 0.50; slope = 1.00; 95% CI, 0.52–1.48), and the Hosmer–Lemeshow test was not statistically significant (*p* = 0.821) (**Figure 2B**). Decision curve analysis indicated that the PNI–ASA nomogram provided greater estimated net benefit than the treat-none, treat-all, and single-predictor strategies across threshold probabilities of 0.00–0.50 (**Figure 2D**). At the optimal threshold of 0.22, the net benefit was 0.18, corresponding to 18 additional correctly identified high-risk patients per 100 individuals without an increase in false-positive ICU admissions.

Panel E of **Figure 2** provides a model-based visualization of the weighted PNI–ASA score and its corresponding predicted probability of postoperative ICU admission. Lower, more negative model scores were associated with lower predicted probabilities, whereas higher, less negative scores were associated with higher predicted probabilities. This panel was intended as a graphical aid for interpreting the final model rather than as a separate integer-based scoring system.

### 3.4 Subgroup analysis

Using the binary risk classification based on the PNI cutoff of 47.225 (**Supplementary Table 6**) and ASA physical status, we performed subgroup analyses to evaluate the consistency of the association across patient strata. As shown in **Figure 3** and **Table 2**, the direction of the association was consistent across all predefined subgroups. All interaction *p* values were >0.05, indicating no statistically significant effect modification by the assessed covariates.

The odds ratios for postoperative ICU admission in high-risk versus low-risk patients ranged from 5.71 to 32.67, and most subgroup-specific associations were statistically significant. However, estimates were imprecise in several small subgroups, including patients with BMI <18.5 or >23.9 kg/m² (OR = 7.87; 95% CI, 0.71–87.25), operative duration <5 hours (OR = 10.00; 95% CI, 0.87–114.74), and pathological complete response (OR = 2.50; 95% CI, 0.39–16.05). The wide confidence intervals and intervals crossing unity indicate limited precision and statistical power. Nevertheless, the consistent direction of the point estimates supported the overall robustness of the observed association.

## 4 Discussion

In this single-center retrospective cohort study of 115 patients with thoracic ESCC who underwent nCIT followed by MIE, 25 (21.7%) required postoperative ICU admission. Across several covariate-adjustment strategies, lower preoperative PNI and ASA class III were consistently associated with postoperative ICU admission. In the final parsimonious PNI–ASA model (**Table 1**, Model 4), each one-point increase in PNI was associated with 16% lower odds of postoperative ICU admission (OR = 0.84; 95% CI, 0.74–0.94; *p* = 0.002), whereas ASA class III was associated with 7.57-fold higher odds than ASA class I/II (OR = 7.57; 95% CI, 2.10–27.20; *p* = 0.002). Operative duration was associated with the outcome in the univariable analysis but was not statistically significant in the most fully adjusted exploratory model. It was therefore considered an intraoperative warning indicator rather than an independent component of the preoperative prediction model.

Lower preoperative PNI was independently associated with postoperative ICU admission, consistent with previous evidence. A meta-analysis by Xue et al. showed that low PNI was associated with adverse outcomes after esophageal cancer surgery (24). Zou et al. reported that a PNI ≤47.38 was significantly associated with higher rates of pulmonary infection and anastomotic leakage among patients receiving nCIT (13), whereas Chen et al. found that a PNI ≤49.6 independently predicted postoperative pneumonia (25). Other studies have demonstrated that low PNI is associated not only with postoperative complications but also with poorer survival in patients with ESCC (26). Nutritional and inflammatory indices have also been shown to predict adverse outcomes, including ICU admission, among unplanned hospitalized oncology patients (19). Our findings extend this evidence by demonstrating the predictive value of PNI for postoperative ICU admission, a severe and resource-intensive clinical endpoint.

ASA class III was independently associated with postoperative ICU admission (OR = 7.57; 95% CI, 2.10–27.20; *p* = 0.002). This finding is consistent with previous studies. Dejen et al. reported a strong association between ASA class III and unplanned ICU admission in a multicenter surgical cohort, whereas Mijiti et al. found that ASA class ≥3 was associated with an increased risk of major postoperative complications in patients with esophageal cancer (17, 18). Our findings suggest that the ASA Physical Status Classification System retains predictive value in the setting of MIE after neoadjuvant immunotherapy. Other simple perioperative risk assessment tools, such as the Surgical Apgar Score, have also been strongly associated with postoperative ICU admission (22), further supporting the clinical utility of readily available risk-stratification measures.

Longer operative duration was associated with postoperative ICU admission in the univariable analysis (OR = 1.01 per minute; 95% CI, 1.00–1.01; *p* = 0.024), and median operative duration was longer among patients requiring ICU admission than among those who did not (379.8 vs. 340.5 min; *p* = 0.018). However, this association was not statistically significant in the fully adjusted multivariable model (Model 3; *p* = 0.102). This attenuation may reflect confounding by case complexity, intraoperative blood loss, or other unmeasured technical factors. Although not an independent predictor, prolonged surgery may still serve as a clinically useful intraoperative warning indicator. Zhang et al. reported a similar finding in a large esophagectomy cohort, in which operative duration was associated with unplanned ICU admission in univariable but not multivariable analysis (11). Thus, although operative duration alone may not independently predict ICU admission, it may alert the surgical team to greater procedural complexity and the potential need for enhanced postoperative monitoring.

Our findings have several clinical implications across the perioperative period. Preoperatively, PNI and ASA class constitute the core of the nomogram and may support early risk stratification. For patients with low PNI or ASA class III, structured prehabilitation programs, including immunonutrition, inspiratory muscle training, and smoking cessation, may be considered before surgery. Intraoperatively, although operative duration was not an independent predictor in the multivariable model, prolonged surgery may indicate heightened risk and prompt enhanced postoperative monitoring. Postoperatively, the nomogram provides individualized risk estimates that may help guide monitoring intensity. Given that respiratory failure occurred in 80% of patients requiring ICU admission, early airway clearance and noninvasive ventilation should be prioritized in high-risk patients. In addition, intraoperative quality-improvement strategies, including adherence to enhanced recovery after surgery ventilation protocols, lung-protective ventilation with a tidal volume of 6–8 mL/kg and appropriate PEEP, goal-directed fluid therapy, and efficient surgical dissection, may help reduce procedural stress.

Generic risk calculators, such as the ACS NSQIP risk tool, have shown limited predictive performance in patients undergoing esophagectomy, with a C-index <0.56 (27). Therefore, improved preoperative risk-assessment tools are needed to support informed decision-making before esophagectomy (16). The model developed in the present study was specifically designed for patients undergoing nCIT followed by MIE and demonstrated acceptable discrimination and favorable calibration.

The PNI cutoff of 47.225 identified in our subgroup analyses was similar to previously reported thresholds in comparable patient populations (**Supplementary Table 8**). Zou et al. reported a cutoff of 47.38 for predicting short-term postoperative complications in patients with ESCC receiving neoadjuvant immunochemotherapy (13), whereas a study of pathological response to neoadjuvant anti-PD-1 therapy plus chemotherapy identified a cutoff of 47.85 (28). Other studies in metastatic or unresectable settings have reported cutoffs ranging from 40.5 to 52.9 (29–32). The convergence of independently derived cutoffs in the range of 47.2–48.0, despite differences in outcome definitions and study designs, provides some reassurance regarding the potential relevance of this range in patients with ESCC receiving neoadjuvant therapy. Nevertheless, the cutoff used in our subgroup analyses was derived from this dataset and requires validation in independent, preferably multicenter, cohorts before clinical implementation.

Several limitations should be acknowledged. First, this was a single-center retrospective cohort study with a relatively small number of postoperative ICU admission events (n = 25). The limited sample size and event count may have reduced the statistical power of the multivariable analyses and precluded meaningful analyses in some subgroups, as reflected by the wide confidence intervals. Second, because of the retrospective design, causal relationships between the identified predictors and postoperative ICU admission cannot be established; the findings should therefore be interpreted as hypothesis-generating associations. Third, the model was developed and internally validated at a single institution with standardized surgical and perioperative protocols. External validation in independent, preferably multicenter, prospective cohorts is required before the nomogram can be generalized to broader clinical settings. Fourth, because of limitations in data availability, we were unable to include detailed intraoperative anesthesia-related variables, such as fluid balance, vasopressor requirements, or intraoperative ventilatory parameters, or dynamic postoperative biomarkers, such as serial C-reactive protein or albumin levels. Incorporating these variables may improve model discrimination in future studies (33). Finally, the dichotomized PNI cutoff of 47.225 used for subgroup stratification was derived from the same dataset using the Youden index and is therefore dataset-specific. This threshold should not be adopted clinically without rigorous external validation. Despite these limitations, the objective and routinely available nature of PNI and ASA class supports the potential clinical utility of the nomogram, pending prospective evaluation.

## 5 Conclusions

In this retrospective cohort, lower preoperative PNI and higher ASA physical status were independently associated with postoperative ICU admission after nCIT followed by MIE. The PNI–ASA nomogram showed acceptable discrimination and calibration and may support preoperative risk stratification. Although operative duration was not independently associated with postoperative ICU admission, it may serve as an intraoperative warning indicator. These findings highlight the potential value of nutritional optimization and careful perioperative assessment. However, given the single-center retrospective design and absence of external validation, the nomogram should be considered hypothesis-generating. Prospective multicenter studies are needed to validate and refine the model before clinical implementation.

## Data Availability

The raw data supporting the conclusions of this article will be made available by the authors, without undue reservation.
