# Supplementary material for: Risk factors for postoperative intensive care unit admission after minimally invasive esophagectomy following neoadjuvant chemoimmunotherapy in patients with esophageal squamous cell carcinoma: a retrospective cohort study
Source: Front Oncol. 2026 Aug 28;16:1917011. doi: 10.3389/fonc.2026.1917011 (PMC13561911; doi:10.3389/fonc.2026.1917011)
Supplement: Supplementary file 1 [file Table1.doc]

Supplementary Table 1. American Society of Anesthesiologists (ASA) Physical Status Classification and distribution of age-adjusted Charlson Comorbidity Index (aCCI) scores

### Part A. ASA Physical Status Classification

| **ASA Class** | **Definition** |
| --- | --- |
| I | A normal healthy patient |
| II | A patient with mild systemic disease, no substantive functional limitations |
| III | A patient with severe systemic disease, substantive functional limitations |
| IV | A patient with severe systemic disease that is a constant threat to life |
| V | A moribund patient who is not expected to survive without the operation |
| VI | A brain-dead patient whose organs are being removed for donor purposes |

Note: The suffix “E” denotes emergency surgery (not applicable in this study).

### Part B. Distribution of age-adjusted Charlson Comorbidity Index (aCCI) scores

| aCCI score | Total (n = 115) | No ICU (n = 90) | ICU (n = 25) | *p* value* |
| --- | --- | --- | --- | --- |
| 1 | 3 (2.6) | 3 (3.3) | 0 (0) | 0.075 |
| 2 | 17 (14.8) | 16 (17.8) | 1 (4) |  |
| 3 | 36 (31.3) | 30 (33.3) | 6 (24) |  |
| 4 | 46 (40.0) | 33 (36.7) | 13 (52) |  |
| 5 | 10 (8.7) | 5 (5.6) | 5 (20) |  |
| 6 | 3 (2.6) | 3 (3.3) | 0 (0) |  |

**p* value from Fisher’s exact test for comparison between ICU and no-ICU groups.

*Note: aCCI was calculated as Charlson Comorbidity Index + age score (age 50-59: +1, 60-69: +2, 70-9: +3, ≥80: +4).

**Supplementary Table 2.** Comparison of laboratory and inflammatory biomarkers between patients with and without postoperative ICU admission.

| Variables | Total (n = 115) | No ICU (n = 90) | ICU (n = 25) | *p* value |
| --- | --- | --- | --- | --- |
| High triglyceride level, n (%) |  |  |  | 0.258 |
| No | 92 (80.0) | 70 (77.8) | 22 (88) |  |
| Yes | 23 (20.0) | 20 (22.2) | 3 (12) |  |
| Hypercholesterolemia, n (%) |  |  |  | 0.511 |
| No | 67 (58.3) | 51 (56.7) | 16 (64) |  |
| Yes | 48 (41.7) | 39 (43.3) | 9 (36) |  |
| Mixed hyperlipidemia, n (%) |  |  |  | 0.458 |
| No | 103 (89.6) | 79 (87.8) | 24 (96) |  |
| Yes | 12 (10.4) | 11 (12.2) | 1 (4) |  |
| Albumin (g/L), Mean ± SD | 41.8 ± 3.5 | 42.4 ± 3.2 | 39.7 ± 3.6 | <0.001 |
| Triglyceride (mmol/L), Median (IQR) | 1.1 (0.9-1.5) | 1.2 (0.9-1.6) | 1.1 (0.9-1.3) | 0.162 |
| Cholesterol (mmol/L), Mean ± SD | 5.0 ± 1.2 | 5.0 ± 1.1 | 4.9 ± 1.5 | 0.513 |
| Fasting blood glucose (mmol/L), Mean ± SD | 5.6 ± 1.3 | 5.5 ± 1.2 | 5.9 ± 1.8 | 0.167 |
| Carcinoembryonic antigen (μg/L), Mean ± SD | 2.3 ± 1.4 | 2.2 ± 1.3 | 2.5 ± 1.7 | 0.371 |
| SII, Median (IQR) | 437.3 (286.1-606.8) | 414.0 (278.0-534.4) | 626.5 (373.2-1067.1) | 0.001 |
| SIRI, Median (IQR) | 0.8 (0.5-1.3) | 0.7 (0.5-1.2) | 1.1 (0.8-1.9) | 0.010 |
| NLR, Median (IQR) | 2.1 (1.4-2.8) | 2.0 (1.3-2.4) | 2.8 (2.0-3.8) | 0.004 |
| dNLR, Mean ± SD | 0.8 ± 0.1 | 0.8 ± 0.1 | 0.8 ± 0.1 | 0.392 |
| PLR, Mean ± SD | 148.6 ± 87.5 | 138.6 ± 85.3 | 184.8 ± 87.1 | 0.019 |
| MLR, Mean ± SD | 0.3 ± 0.2 | 0.3 ± 0.2 | 0.4 ± 0.2 | 0.063 |
| NPR, Mean ± SD | 0.0 ± 0.0 | 0.0 ± 0.0 | 0.0 ± 0.0 | 0.533 |
| NMLR, Median (IQR) | 2.3 (1.6-3.0) | 2.2 (1.5-2.7) | 3.1 (2.3-4.1) | 0.003 |
| PIV, Median (IQR) | 191.0 (112.6-300.9) | 154.0 (104.4-257.2) | 259.6 (193.5-465.9) | 0.003 |
| HALP Score, Mean ± SD | 41.4 ± 21.4 | 44.1 ± 21.7 | 31.7 ± 17.3 | 0.010 |

Abbreviations: ICU, intensive care unit; SD, standard deviation; IQR, interquartile range; SII, systemic immune‑inflammation index (platelet × neutrophil / lymphocyte); SIRI, systemic inflammation response index (neutrophil × monocyte / lymphocyte); NLR, neutrophil‑to‑lymphocyte ratio; dNLR, derived NLR (neutrophil / (white blood cell - neutrophil)); PLR, platelet‑to‑lymphocyte ratio; MLR, monocyte‑to‑lymphocyte ratio; NPR, neutrophil‑to‑platelet ratio; NMLR, neutrophil × monocyte‑to‑lymphocyte ratio; PIV, pan‑immune‑inflammation value (neutrophil × platelet × monocyte / lymphocyte); HALP, hemoglobin, albumin, lymphocyte, platelet score (hemoglobin × albumin × lymphocyte / platelet).

Supplementary Table 3. Comparison of the area under the ROC curve (AUC) for PNI+ASA, ASA, and PNI in predicting postoperative ICU admission

| **Marker** | **AUC (95% CI)** | **P value*** | **DeLong test (vs. PNI+ASA)** |
| --- | --- | --- | --- |
| PNI+ASA | 0.810 (0.709-0.912) | 0.002 | — |
| ASA | 0.647 (0.547-0.746) | 0.001 | *p* = 0.003 |
| PNI | 0.717 (0.586-0.846) | 0.001 | *p* = 0.178 |

**p* value for test of AUC = 0.5 (null hypothesis of no discrimination).

Note: DeLong test was used to compare the AUC of PNI+ASA with ASA/PNI. The analysis was performed using R software. PNI, prognostic nutritional index; ASA, American Society of Anesthesiologists classification; ICU, intensive care unit.
