# Supplementary material for: Risk factors for postoperative intensive care unit admission after minimally invasive esophagectomy following neoadjuvant chemoimmunotherapy in patients with esophageal squamous cell carcinoma: a retrospective cohort study
Source: Front Oncol. 2026 Aug 28;16:1917011. doi: 10.3389/fonc.2026.1917011 (PMC13561911; doi:10.3389/fonc.2026.1917011)
Supplement: Supplementary file 2 [file Table2.doc]

Supplementary Table 1. American Society of Anesthesiologists (ASA) Physical Status Classification and distribution of age-adjusted Charlson Comorbidity Index (aCCI) scores

### Part A. ASA Physical Status Classification

| ASA Class | Definition |
| --- | --- |
| I | A normal healthy patient |
| II | A patient with mild systemic disease, no substantive functional limitations |
| III | A patient with severe systemic disease, substantive functional limitations |
| IV | A patient with severe systemic disease that is a constant threat to life |
| V | A moribund patient who is not expected to survive without the operation |
| VI | A brain-dead patient whose organs are being removed for donor purposes |

Note: The suffix “E” denotes emergency surgery (not applicable in this study).

### Part B. Distribution of age-adjusted Charlson Comorbidity Index (aCCI) scores

| aCCI score | Total (n = 115) | No ICU (n = 90) | ICU (n = 25) | *p* value* |
| --- | --- | --- | --- | --- |
| 1 | 3 (2.6) | 3 (3.3) | 0 (0) | 0.075 |
| 2 | 17 (14.8) | 16 (17.8) | 1 (4) |  |
| 3 | 36 (31.3) | 30 (33.3) | 6 (24) |  |
| 4 | 46 (40.0) | 33 (36.7) | 13 (52) |  |
| 5 | 10 (8.7) | 5 (5.6) | 5 (20) |  |
| 6 | 3 (2.6) | 3 (3.3) | 0 (0) |  |

**p* value from Fisher’s exact test for comparison between ICU and no-ICU groups.

*Note: aCCI was calculated as Charlson Comorbidity Index + age score (age 50-59: +1, 60-69: +2, 70-79: +3, ≥80: +4).

Supplementary Table 2. Comparison of laboratory and inflammatory biomarkers between patients with and without postoperative ICU admission.

| Variables | Total (n = 115) | No ICU (n = 90) | ICU (n = 25) | *p* value |
| --- | --- | --- | --- | --- |
| High triglyceride level, n (%) |  |  |  | 0.258 |
| No | 92 (80.0) | 70 (77.8) | 22 (88) |  |
| Yes | 23 (20.0) | 20 (22.2) | 3 (12) |  |
| Hypercholesterolemia, n (%) |  |  |  | 0.511 |
| No | 67 (58.3) | 51 (56.7) | 16 (64) |  |
| Yes | 48 (41.7) | 39 (43.3) | 9 (36) |  |
| Mixed hyperlipidemia, n (%) |  |  |  | 0.458 |
| No | 103 (89.6) | 79 (87.8) | 24 (96) |  |
| Yes | 12 (10.4) | 11 (12.2) | 1 (4) |  |
| Albumin (g/L), Mean ± SD | 41.8 ± 3.5 | 42.4 ± 3.2 | 39.7 ± 3.6 | <0.001 |
| Triglyceride (mmol/L), Median (IQR) | 1.1 (0.9-1.5) | 1.2 (0.9-1.6) | 1.1 (0.9-1.3) | 0.162 |
| Cholesterol (mmol/L), Mean ± SD | 5.0 ± 1.2 | 5.0 ± 1.1 | 4.9 ± 1.5 | 0.513 |
| Fasting blood glucose (mmol/L), Mean ± SD | 5.6 ± 1.3 | 5.5 ± 1.2 | 5.9 ± 1.8 | 0.167 |
| Carcinoembryonic antigen (μg/L), Mean ± SD | 2.3 ± 1.4 | 2.2 ± 1.3 | 2.5 ± 1.7 | 0.371 |
| SII, Median (IQR) | 437.3 (286.1-606.8) | 414.0 (278.0-534.4) | 626.5 (373.2-1067.1) | 0.001 |
| SIRI, Median (IQR) | 0.8 (0.5-1.3) | 0.7 (0.5-1.2) | 1.1 (0.8-1.9) | 0.010 |
| NLR, Median (IQR) | 2.1 (1.4-2.8) | 2.0 (1.3-2.4) | 2.8 (2.0-3.8) | 0.004 |
| dNLR, Mean ± SD | 0.8 ± 0.1 | 0.8 ± 0.1 | 0.8 ± 0.1 | 0.392 |
| PLR, Mean ± SD | 148.6 ± 87.5 | 138.6 ± 85.3 | 184.8 ± 87.1 | 0.019 |
| MLR, Mean ± SD | 0.3 ± 0.2 | 0.3 ± 0.2 | 0.4 ± 0.2 | 0.063 |
| NPR, Mean ± SD | 0.0 ± 0.0 | 0.0 ± 0.0 | 0.0 ± 0.0 | 0.533 |
| NMLR, Median (IQR) | 2.3 (1.6-3.0) | 2.2 (1.5-2.7) | 3.1 (2.3-4.1) | 0.003 |
| PIV, Median (IQR) | 191.0 (112.6-300.9) | 154.0 (104.4-257.2) | 259.6 (193.5-465.9) | 0.003 |
| HALP Score, Mean ± SD | 41.4 ± 21.4 | 44.1 ± 21.7 | 31.7 ± 17.3 | 0.010 |

Abbreviations: ICU, intensive care unit; SD, standard deviation; IQR, interquartile range; SII, systemic immune‑inflammation index (platelet × neutrophil / lymphocyte); SIRI, systemic inflammation response index (neutrophil × monocyte / lymphocyte); NLR, neutrophil‑to‑lymphocyte ratio; dNLR, derived NLR (neutrophil / (white blood cell - neutrophil)); PLR, platelet‑to‑lymphocyte ratio; MLR, monocyte‑to‑lymphocyte ratio; NPR, neutrophil‑to‑platelet ratio; NMLR, neutrophil × monocyte‑to‑lymphocyte ratio; PIV, pan‑immune‑inflammation value (neutrophil × platelet × monocyte / lymphocyte); HALP, hemoglobin, albumin, lymphocyte, platelet score (hemoglobin × albumin × lymphocyte / platelet).

Supplementary Table 3. Multicollinearity assessment of variables included in the multivariable logistic regression models

| Variable | VIF |
| --- | --- |
| Sex (Male vs. Female) | 1.360 |
| Age group (＜65 years vs. ≥65 years) | 1.142 |
| BMI (continuous) | 1.119 |
| Hypertension(No vs. Yes） | 1.603 |
| Diabetes(No vs. Yes） | 1.288 |
| Immunotherapy drug (PD-1 vs. PD-L1) | 1.442 |
| Neoadjuvant cycles (2 cycles vs. 3/4 cycles) | 1.266 |
| ASA class (I/II vs. III) | 1.293 |
| PNI (continuous) | 1.148 |
| FEV1 (L) | 1.444 |
| Surgical duration (min) (continuous) | 1.303 |
| Intraoperative blood loss (＜500ml vs. ≥500ml） | 1.211 |

Note: All VIF values are < 2, indicating no multicollinearity (conventional thresholds: VIF > 10 = severe; VIF > 5 = moderate).

Supplementary Table 4. Comparison of the areas under the receiver operating characteristic curves for the PNI–ASA model, ASA alone, and PNI alone in predicting postoperative ICU admission

| Marker | AUC (95% CI) | *P* value* | DeLong test (vs. PNI+ASA) |
| --- | --- | --- | --- |
| PNI–ASA model | 0.810 (0.709-0.912) | 0.002 | — |
| ASA alone | 0.647 (0.547-0.746) | 0.001 | *p* = 0.003 |
| PNI alone | 0.717 (0.586-0.846) | 0.001 | *p* = 0.178 |

**p* value for test of AUC = 0.5 (null hypothesis of no discrimination).

Note: The DeLong test was used to compare the AUC of the PNI–ASA model with those of ASA alone and PNI alone. The analysis was performed using R software. PNI, prognostic nutritional index; ASA, American Society of Anesthesiologists classification; ICU, intensive care unit.

Supplementary Table 5. Bootstrap internal validation of the PNI–ASA model

| Performance metric | Apparent performance | Optimism | Optimism-corrected performance |
| --- | --- | --- | --- |
| AUC (95% CI) | 0.810 (0.709–0.912) | 0.029 | 0.781 (0.674–0.888) |
| Calibration slope | 1 | 0.06 | 0.94 |
| Calibration intercept | -0.00 | 0.02 | -0.02 |
| Brier score | 0.158 | 0.014 | 0.172 |

Bootstrap iterations = 1,000. Abbreviation: AUC, area under the receiver operating characteristic curve. For the Brier score, the optimism-corrected value was obtained by adding the estimated optimism because lower values indicate better performance.

Supplementary Table 6. Diagnostic performance of PNI cutoff (47.225) for predicting postoperative ICU admission

| Metric | Value | 95% CI |
| --- | --- | --- |
| Sensitivity | 68.00% | 46.6–85.1% |
| Specificity | 78.90% | 69.0 – 86.6% |
| Positive predictive value | 47.20% | 30.9 – 63.5% |
| Negative predictive value | 89.90% | 83.2 – 96.5% |
| Youden index | 0.469 | — |

CI, confidence interval. Sensitivity and specificity were calculated using the Clopper‑Pearson exact method; PPV and NPV using the standard binomial method.

Supplementary Table 7. Timing, indications, and postoperative complications for ICU-admitted patients

| Patient | POD* | Indication for ICU admission | Complications present at ICU admission | Complications developed during ICU stay |
| --- | --- | --- | --- | --- |
| 1 | 3 | Pneumonia | Pneumonia | Respiratory failure |
| 2 | 0 | Difficult extubation + pneumonia | Respiratory failure, pneumonia | — |
| 3 | 3 | Respiratory failure + pneumonia | Respiratory failure, pneumonia | — |
| 4 | 2 | Pulmonary embolism | Pulmonary embolism | — |
| 5 | 3 | Pneumonia | Pneumonia | — |
| 6 | 1 | Respiratory failure | Respiratory failure | — |
| 7 | 3 | Respiratory failure | Respiratory failure | — |
| 8 | 3 | Respiratory failure + pneumonia | Respiratory failure, pneumonia | — |
| 9 | 3 | Pneumonia | Pneumonia | — |
| 10 | 0 | Respiratory failure | Respiratory failure | — |
| 11 | 4 | Pneumonia | Pneumonia | — |
| 12 | 1 | Respiratory failure | Respiratory failure | — |
| 13 | 3 | Respiratory failure + pneumonia | Respiratory failure, pneumonia | — |
| 14 | 27† | Respiratory failure + pneumonia | Respiratory failure, pneumonia | — |
| 15 | 0 | Difficult extubation | Respiratory failure | — |
| 16 | 0 | Respiratory failure + pneumonia | Respiratory failure, pneumonia | — |
| 17 | 3 | Respiratory failure + pneumonia | Respiratory failure, pneumonia | — |
| 18 | 0 | Respiratory failure + pneumonia | Respiratory failure, pneumonia | — |
| 19 | 0 | Difficult extubation | Respiratory failure | — |
| 20 | 0 | Respiratory failure + pneumonia | Respiratory failure, pneumonia | — |
| 21 | 0 | Difficult extubation + pneumonia | Respiratory failure, pneumonia | — |
| 22 | 0 | Difficult extubation + pneumonia | Respiratory failure, pneumonia | — |
| 23 | 1 | Acute ischemic intestinal necrosis | Acute ischemic intestinal necrosis, hypovolemic shock | Respiratory failure |
| 24 | 0 | Hyperkalemia | Hyperkalemia | — |
| 25 | 0 | Difficult extubation | Respiratory failure | — |

POD, postoperative day (day of surgery = POD 0).

† This patient developed respiratory failure secondary to cervical anastomotic leakage, representing the latest ICU admission on POD 27.

Supplementary Table 8. Comparison of PNI thresholds reported in esophageal cancer literature

| Study | Population | Sample size | PNI cutoff | Outcome |
| --- | --- | --- | --- | --- |
| Present study | ESCC, nCIT + MIE | 115 | 47.225 | Postoperative ICU admission |
| Zou et al. (2024) | ESCC, nCIT | 77 | 47.38 | Postoperative complications |
| Song et al. (2025) | ESCC, nCIT | 116 | 47.85 | Good response |
| Chen & Li (2025) | Esophageal cancer, esophagectomy | 200 | 49.6 | Postoperative pneumonia |
| Zheng et al. (2022) | Resectable ESCC | 165 | 43.85 | Overall survival |
| Zhang et al. (2022) | ESCC | 271 | 49.1 | 5‑year survival |
| Yoshino et al. (2025) | Metastatic ESCC, ICI monotherapy | 109 | 40.5 | Overall survival |
| Chantharakhit et al. (2022) | Unresectable EC | 170 | 52.9 | Overall survival |

Abbreviations: ESCC, esophageal squamous cell carcinoma; nCIT, neoadjuvant chemoimmunotherapy; MIE, minimally invasive esophagectomy; LA‑ESCC, locally advanced esophageal squamous cell carcinoma; ICI, immune checkpoint inhibitor; EC, esophageal cancer; pCR, pathological complete response; NLR, neutrophil‑to‑lymphocyte ratio.
